# Four terpene synthases contribute to the generation of chemotypes in tea tree (*Melaleuca alternifolia*)

**DOI:** 10.1186/s12870-017-1107-2

**Published:** 2017-10-04

**Authors:** Amanda Padovan, Andras Keszei, Yasmin Hassan, Sandra T. Krause, Tobias G. Köllner, Jörg Degenhardt, Jonathan Gershenzon, Carsten Külheim, William J. Foley

**Affiliations:** 1 Division of Ecology and Evolution, Research School of Biology, The Australian National University, Canberra, 2601 Australia; 2 Institute of Pharmacy, Martin Luther University, Hoher Weg 8, 06120 Halle, Germany; 3 Max Planck Institute for Chemical Ecology, Hans-Knöll-Strasse 8, 07745 Jena, Germany

**Keywords:** Terpene, Essential oil, Functional characterisation, Gene expression, Natural population

## Abstract

**Background:**

Terpene rich leaves are a characteristic of Myrtaceae. There is significant qualitative variation in the terpene profile of plants within a single species, which is observable as “chemotypes”. Understanding the molecular basis of chemotypic variation will help explain how such variation is maintained in natural populations as well as allowing focussed breeding for those terpenes sought by industry. The leaves of the medicinal tea tree, *Melaleuca alternifolia*, are used to produce terpinen-4-ol rich tea tree oil, but there are six naturally occurring chemotypes; three cardinal chemotypes (dominated by terpinen-4-ol, terpinolene and 1,8-cineole, respectively) and three intermediates. It has been predicted that three distinct terpene synthases could be responsible for the maintenance of chemotypic variation in this species.

**Results:**

We isolated and characterised the most abundant terpene synthases (TPSs) from the three cardinal chemotypes of *M. alternifolia*. Functional characterisation of these enzymes shows that they produce the dominant compounds in the foliar terpene profile of all six chemotypes. Using RNA-Seq, we investigated the expression of these and 24 additional putative terpene synthases in young leaves of all six chemotypes of *M. alternifolia*.

**Conclusions:**

Despite contributing to the variation patterns observed, variation in gene expression of the three TPS genes is not enough to explain all variation for the maintenance of chemotypes. Other candidate terpene synthases as well as other levels of regulation must also be involved. The results of this study provide novel insights into the complexity of terpene biosynthesis in natural populations of a non-model organism.

**Electronic supplementary material:**

The online version of this article (10.1186/s12870-017-1107-2) contains supplementary material, which is available to authorized users.

## Background

Intra-specific variation in plant phenotypes can have profound ecological consequences [1–3]. In particular, variation in plant specialised metabolites influences herbivores as selective agents on the survival of some individuals over others [4, 5], and even dictates the success of biological control programmes for weeds [6, 7].

Understanding how intra-specific variation in plant chemical profiles arises at the molecular level would help explain how it is maintained in natural populations [8, 9]. Quantitative variation in specialised metabolites is the norm and suggests that there are multiple selective agents operating on these traits [10]. In contrast, it is less clear how discontinuous or “chemotypic” variation is maintained in long-lived plants such as forest trees and it remains difficult to demonstrate exactly what selective agents are influential over the many years that the tree may grow [11]. Characterising the genes responsible and the factors that control their expression remains the first step to resolving this question.

Medicinal tea tree (*Melaleuca alternifolia* (Maiden & Betche) Cheel: Family Myrtaceae) is an excellent system to examine chemotypic variation. Tea tree is a long-lived woody plant that occurs in six distinct, foliar terpene chemotypes: three cardinal chemotypes dominated by terpinolene, 1,8-cineole and terpinen-4-ol respectively, and three intermediates between these [12, 13]. The chemotypes can occur in pure natural stands but some sites can contain mixtures of up to five chemotypes. Only one of these chemotypes yields a medicinally valuable essential oil dominated by the monoterpene terpinen-4-ol and an industry is focussed on the cultivation of this chemotype [14]. Tea tree oil is widely used in products for personal care as well as having household, agricultural and veterinary applications. It shows significant antifungal and antibacterial activity in vivo [15] and has promising effects on skin tumours [16].

Enhancing the foliar concentration of medicinally active terpinen-4-ol and reducing the concentrations of 1,8-cineole and *d-*limonene is a major aim of breeding programmes [14] and thus knowing the genes that underlie these traits would be invaluable for enhancing breeding using molecular markers. A putative monoterpene synthase was isolated previously from *M. alternifolia* [17], but further analysis showed that the product of this enzyme is isoprene [18, 19].

Studies of the foliar chemistry of *M. alternifolia* led to the hypothesis that only three distinct terpene synthases are responsible for the biosynthesis of over 80% of the leaf terpenes [13] with control of their contributions of each to the final oil profile likely dependent on genomic, transcriptomic or proteomic differences. Studies in other plants have shown that transcriptional level control of terpene synthases is most common. For example, Crocoll et al. found that transcript abundance of terpene synthases was correlated with variations in terpenes in oregano [20] and Irmisch et al. found that transcriptional differences in five sesquiterpene synthases explained the pattern of accumulation of terpenes in different parts of chamomile [21].

In this study, we aim (1) to isolate and functionally characterise terpene synthases that produce terpinen-4-ol, terpinolene, and 1,8-cineole, respectively; and (2) to determine the expression of these genes in naturally occurring individuals from each of six chemotypes.

## Methods

This study was carried out in two parts. Firstly, we amplified and characterised the genes responsible for the production of the terpenes that dominate the cardinal chemotypes of *M. alternifolia*. In the second part of this study we investigated the expression of terpene synthases in natural populations containing up to five chemotypes per population and compared the gene expression to terpene variation. All plant material was collected from private properties with the express permission of the land owners.

### Part 1: Amplification and characterisation of terpene synthases

#### Plant material

Young leaf (ca. 5 g fresh weight) was collected from five mature trees at nine sites across the natural geographic range of *M. alternifolia* [13]. We chose trees that were at least 100 m apart to avoid collecting from related trees [22] and the location of each tree was recorded. Samples were snap frozen in liquid nitrogen and stored at −80 °C to ensure that we had samples suitable for RNA extraction from trees belonging to each of the known chemotypes.

#### Extraction of nucleic acid

We extracted total RNA from young leaf ground in liquid nitrogen using the RNeasy Plant Micro kit (Qiagen, Australia). We complemented the lysis buffer with polyvinyl pyrrolidine and sodium isoascorbate (Suzuki et al. 2003). This combination enhanced RNA extraction in all plants except those of Chemotype 2, where the addition of sodium isoascorbate inhibited RNA extraction. Thus, we repeated those extractions without this adjuvant (data not shown).

#### Isolation, identification and characterisation of terpene synthases

We used 3′ ‘rapid amplification of complimentary ends’ or RACE reactions to obtain partial transcripts containing the terpene synthase DDxxD motif using the degenerate ‘DDXYDfx’ primer and T_35_VN previously used to successfully isolate terpene synthases from 21 species of Myrtaceae [19]. We ligated the amplification products into pGEMT Easy or pCR2.1^TOPO^ cloning vectors, and sequenced the inserts from the M13 priming sites using BigDye v. 3.1 on an ABI 3130 capillary sequencer. Sequence information from the most abundant transcripts in each chemotype was used to design primers for upstream amplification. We used the SMART 5’RACE kit to amplify the 5′ ends of the identified genes, and obtained sequence information using the reaction conditions described for 3’RACE. Following the assembly of the 3′ and 5′ contigs, we designed primers to obtain full-length cDNA clones. We used Primer3 [23] to design primers, and used these to amplify clones encoding pseudo-mature proteins for characterisation.

PCR was performed using Advantage 2 polymerase mix (BD Biosciences, CA, USA). To amplify the two 1,8-cineole synthases *MaTPS-CinA* and *MaTPS-CinB*, we used 5′-CTTCACAATGGCTCTTCCTGCTTTGTCC and 5′-TGTCCAAGCACCGTCAATAG; to amplify the terpinolene synthase *MaTPS-Tln* we used 5′-TTTCCCAATGGCTCTTCCATCAC and 5′-AACATCGAAGGCTCAGTCCGAAAGC; and to amplify the sabinene hydrate synthase *MaTPS-SaH* we used 5′-CGGGGACAACAAACTTCACAATGGC and 5′-ACGAAGCTGTCCAAGCACCGTC. The resulting PCR products were directly inserted as *Bsp*MI fragments into the expression vector pASK-IBA7 (IBA GmbH, Göttingen, Germany). Expression and partial purification of the recombinant protein followed the procedure described in Köllner et al. [24]. To determine the catalytic activity of the recombinant proteins, enzyme assays containing 50 μl of the bacterial extract and 50 μl assay buffer (10 mM MOPSO [pH 7.0], 1 mM dithiothreitol, 10% [*v*/v] glycerol) with 10 μM substrate (geranyl pyrophosphate: GPP (Echelon Biosciences, UT, USA) and (*E,E*)-farnesyl pyrophosphate: FPP, respectively), a divalent metal cofactor (10 mM MgCl_2_), 0.2 mM Na_2_WO_4_ and 0.1 mM NaF in a Teflon-sealed, screw-capped 1 ml GC glass vial were performed. A solid phase microextraction (SPME) fibre consisting of 100 μm polydimethylsiloxane (SUPELCO, PA, USA) was placed into the headspace of the vial for a 30-min incubation at 30 °C. For analysis of the adsorbed reaction products, the SPME fibre was directly inserted into the injector of the gas chromatograph.

A Shimadzu model 2010 gas chromatograph was employed with the carrier gas He at 1 ml·min^−1^, splitless injection (injector temperature: 220 °C, injection volume: 1 μl), a Chrompack CP-SIL-5 CB-MS column ((5%-phenyl)-methylpolysiloxane, 25 m × 0.25 mm i.d. × 0.25 μm film thickness, Varian, CA, USA) and a temperature program from 50 °C (3-min hold) at 6 °C min^−1^ to 180 °C (1-min hold). The coupled mass spectrometer was a Shimadzu model QP2010Plus with a quadrupole mass selective detector, transfer line temperature: 230 °C, source temperature: 230 °C, quadrupole temperature: 150 °C, ionization potential: 70 eV and a scan range of 50–300 amu. Compounds produced by MaTPS-SaH, MaTPS-Cin and MaTPS-Tln were identified by comparison of mass spectra and retention times to those of authentic standards [25] or using the Wiley library of mass spectra.

Chiral GC-MS analysis of the products of MaTPS-SaH was performed on the same instrument using a Rt™-βDEXsm-column (Restek, Bad Homburg, Germany) and a temperature program from 50 °C (2-min hold) at 2 °C min^−1^ to 220 °C (1-min hold). Enantiomers were identified according to their elution order as described by Larkov et al. [26].

#### Enzyme kinetics

A crude extract of each enzyme (30 μl) was incubated with 0.25 mM manganese and 5 μM ^3^H–labeled GPP (American Radiolabeled Chemicals, MO, USA) for different times within a range of 5–30 min to determine the linear phase of the enzymes (results: MaTPS-SaH: 15 min; MaTPS-Cin: 10 min; MaTPS-Tln: 15 min). For the determination of the substrate K_*m*_ values, the enzymes were incubated with 0.25 mM manganese and ^3^H–GPP in a range of 1–30 μM. For MaTPS-Tln, the GPP concentration was increased up to 100 μM to determine the saturation region.

For the determination of the cofactor K_*m*_ values of MaTPS-Tln, the enzyme was incubated with 5 μM ^3^H–labeled GPP and magnesium within a range of 0.5–50 mM or manganese in a range of 0.01–1 mM.

All assays were overlaid with 1 ml pentane and incubated at 30 °C for 10 or 15 min, depending on the linear phase. The assays were stopped by shaking at 1400 rpm for 2 min to partition terpene volatiles in the solvent phase. 500 μl pentane were mixed with 2 ml of scintillation cocktail (RotiSzint2200, Roth, Karlsruhe, Germany) and activity was measured in a scintillation counter (LS 6500, Beckman Coulter Inc., Krefeld, Germany). All assays were performed in triplicate. The amount of substrate needed to achieve half of the maximum reaction velocity, or K_*m*_ values, were determined using the Lineweaver-Burke method.

### Part 2: Expression of terpene synthases in young leaf of *Melaleuca alternifolia*

#### Plant material

We collected young leaf from trees labelled in Part 1, in November 2015. Since these were wild populations growing in natural conditions, some of the trees could not be found again (e.g. tree death or label overgrowth) and therefore some additional trees were sampled. The terpene profile of each of the 92 samples collected was determined and chemotypes were assigned (according to Keszei et al. [13]). We collected three sub-samples from each tree whilst in the field:

1. Approximately 3 g of young leaf was collected for RNA extraction. This sample was put into a labelled paper envelope and immediately snap frozen in liquid nitrogen. Upon return to the lab, it was stored at −80 °C until extraction of RNA.
2. Approximately 0.5 g of young leaf was collected for terpene analysis. This sample was put directly into about 5 ml of ethanol (including 0.25 g·l^−1^ tetradecane as an internal standard) of predetermined weight. The vials were weighed again at the end of the day, to record the exact weight of the leaf.
3. An additional 0.5 g of young leaf was collected to determine the fresh weight to dry weight ratio. This sample was put in a labelled paper envelope and stored above ice until the end of the day, when it was weighed and stored at room temperature. Upon returning to the lab, these samples were oven dried at 40 °C to constant mass and the dry weight was recorded.

#### Terpene analysis

Foliar terpenes were analysed as described in Padovan et al. [3]. Briefly, terpenes were separated using gas chromatography on an Agilent 6890 GC using an Alltech AT-35 (35% phenyl, 65% dimethylpolyoxylane) column (Alltech, DE, USA). The column was 60 m long and He was used as the carrier gas. One μl of the ethanol extract was injected at 250 °C at a 1:25 split ratio. The total elution time was 25 min. The components of the solvent extract were detected using an Agilent 5973 Mass Spectrometer. Peaks were identified by comparisons of mass spectra to reference spectra in the National Institute of Standards and Technology library (Agilent Technologies, IL, USA) and major peaks were verified by reference to authentic standards [13].

We identified 18 samples corresponding to three from each of the six chemotypes, to use with gene expression analysis, by comparison with the original samples in Keszei et al. [13].

#### RNA extraction and transcriptome sequencing

RNA extraction and transcriptome sequencing were carried out as described by Padovan et al. [27]. Briefly, the samples were ground to a fine powder in a mortar and pestle under liquid nitrogen. Total RNA was extracted using the Spectrum Total RNA Kit as per the manufacturer’s instructions (Sigma Aldrich, MO, USA). We then used the Illumina TruSeq RNA library preparation kit as per manufacturer’s instructions (Illumina Inc., CA, USA). The libraries were validated on a Bioanalyzer 2100 (Agilent Techonolgies, CA, USA), pooled and sequenced on two lanes of the Illumina HiSeq 2000 platform at the Biomolecular Resource Facility at the Australian National University, using a 150 bp paired-end run (all sequences were uploaded to the SRA database under the Bioproject ID: PRJNA388506).

#### Data analysis

After sequencing, raw reads were separated by barcode and filtered by quality using the HiSeq 2000 software. We then checked the raw reads for quality and adapter contamination using fastqc [28]. FLEXBAR [29] was used to remove low quality bases and remaining sequencing adaptors using the following parameters; Removal of Illumina sequencing adapters with a minimum overlap of 6, threshold of 2, trimming at any end and relaxed adapter option; minimum quality of 30, maximum number of uncalled bases of 1, and minimum remaining read length of 40.

For each of the three cardinal chemotypes, the individual with the highest amount of raw data was selected for de-novo assembly using the Trinity software [30] with default settings. Next, we created a single consensus transcriptome by clustering the transcripts of each of the three samples using CD-HIT-EST with a threshold of 0.94 [31]. At this threshold, the most similar terpene synthase genes were maintained as separate contigs. We then searched the consensus transcriptome for expressed terpene synthase genes and discovered 27.

Each sample was then mapped against the consensus transcriptome using BWA-mem [32] with standard parameters, producing BAM alignments that were sorted and indexed with SAMtools [33]. For each sample, the number of reads mapping to each contig were counted using Qualimap v 2.1.2 comp-counts [34] with the proportional method but with otherwise standard parameters.

We compared two approaches for counting reads mapped to the characterised genes since the terpene synthases are a very large gene family [35] and two of the characterised genes have 98% nucleic acid identity (Additional file 1: Table S1). Approach 1 used the read counts generated from Qualimap comp counts to calculate ‘fragments per kilobase of transcript per million mapped reads’ or FPKM values. Approach 2 corrected the read count based on three (sabinene hydrate synthase) or four (cineole and terpinolene synthases) amino acids that reliably differentiate these three terpene synthase gene sequences before calculating FPKM values. These amino acids are important in determining the product profile of the enzymes [36]. The second approach yielded much lower values, but the relative expression of the three genes was the same in both approaches, so we decided to proceed with the more traditional first approach.

We used sparse partial least squares analysis (sPLS) to explore the associations between expression of the terpene synthases (*N* = 27) and the terpene composition of the leaf, using the R package mixOmics [37]. The gene expression matrix (log transformed FPKM values) was used to explain variation in the terpene matrix using the sPLS regression mode. We analysed the association between genes and terpenes with a correlation plot of the selected variables, using the first two components of the sPLS. In this plot, variables from each matrix are placed on a circular correlation plot. Those variables that are most strongly associated are plotted in the same direction, and the greater the distance from the origin the stronger the correlation. We also prepared heatmaps to show correlations between terpene and gene dataset using the similarity matrices based on the selected variables by the sparse method and the loading vectors for the first three components of the PLS.

### Relationship between the terpene synthases of *M. alternifolia*

We manually aligned the three characterised terpene synthase sequences, the 24 putative terpene synthases found in the transcriptome data generated here and the 113 terpene synthases found in the *Eucalyptus grandis* genome [38, 39] in BioEdit [40]. The alignments were improved on the Clustal Omega server, using default settings [41–43] before phylogenetic trees were generated using the PhyML server, with 1000 bootstraps and using the JTT + I + F + G substitution model [44, 45]. The tree was visualised in FigTree v1.4.3 [46].

## Results

### Terpene synthases which produce terpinen-4-ol, terpinolene, and 1,8-cineole (part 1)

We isolated and sequenced the most abundant foliar monoterpene synthases from leaf of plants belonging to each of the three cardinal chemotypes (Chemotypes 1, 2 and 5) of *M. alternifolia*. Besides obtaining a full-length sequence of a previously described cDNA fragment (*MATPS-SAH*: [19] from the terpinen-4-ol Chemotype 1, we identified three new full-length *M. alternifolia* monoterpene synthase sequences. *MaTPS-CinA* is the characteristic transcript from the 1,8-cineole-dominated Chemotype 5, *MaTPS-CinB* and *MaTPS-Tln* were isolated from the terpinolene-rich Chemotype 2. All four transcripts encode proteins having N-terminal chloroplast targeting sequences, and show between 78 and 98% DNA sequence identity (68–96% amino acid sequence identity) (Additional file 1: Table S1).

**Fig. 1:**
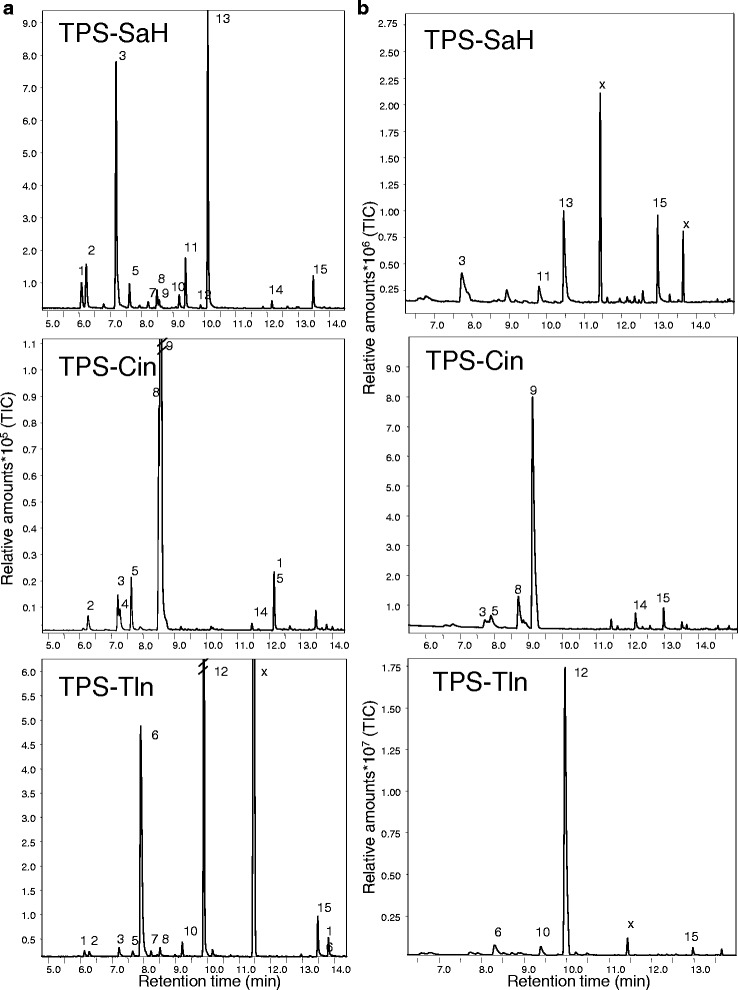
Products of *Melaleuca alternifolia* terpene synthases. The enzymes were expressed in *E. coli*, extracted, and incubated with the substrate GPP. Terpene products were extracted with SPME and analyzed by GC-MS on a EC5-column (**a**) or a AT-35-column (**b**). The products were: 1: α-thujene, 2: α-pinene, 3: sabinene, 4: β-pinene, 5: myrcene, 6: α-phellandrene, 7: α-terpinene 8: limonene, 9: 1,8-cineole, 10: γ-terpinene, 11: (*E*)-sabinene hydrate, 12: terpinolene, 13: (*Z*)-sabinene hydrate, 14: α-terpineol, 15: geraniol (substrate artifact), 16: geranial (substrate artifact), x: silica contamination

**Fig. 2:**
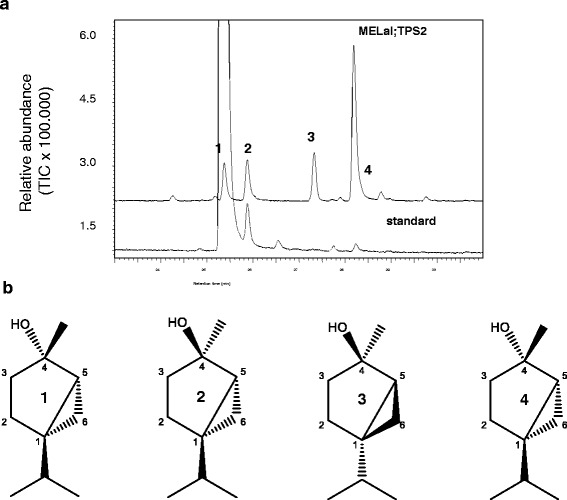
**a** Chiral GC MS analysis of sabinene hydrate formed by the recombinant enzyme, MaTPS-SaH. The elution order of the enantiomers is 1: (*1R,4S,5S*)-(*E*)-sabinene hydrate, 2: (*1S,4R,5R*)-(*E*)-sabinene hydrate, 3**:** (*1R,4R,5S*)-(*Z*)-sabinene hydrate, 4**:** (*1S,4S,5R*)-(*Z*)-sabinene hydrate. **b** The chemical structures of the compounds produced by the recombinant enzyme, MaTPS-SaH

Functional characterisation of the N-terminal truncated MaTPS enzymes showed that each of the proteins was able to convert GPP into different monoterpenes (Fig. 1). FPP, however, was not accepted as substrate (data not shown). The major GPP-derived product of MaTPS-SaH is (*Z*)-sabinene hydrate, which readily converts (non-enzymatically) to terpinen-4-ol in planta [47, 48]; α-terpinene, γ-terpinene, and terpinolene were also produced by this enzyme. MaTPS-CinA and MaTPS-CinB have 1,8-cineole as their major product, but also produce sabinene, limonene, and α-terpineol. MaTPS-Tln produces terpinolene as its major product and appears to be the most product-specific of the four terpene synthases. Chiral analysis of the sabinene hydrate formed by MaTPS-SaH revealed that over 95% of the (*Z*)-form was the (*1R*,*4R*,*5S*)--enantiomer whereas (*E*)-sabinene hydrate was produced in a racemic mixture containing both the (*1R*,*4S*,*5S*)-enantiomer and the (*1S*,*4R*,*5R*)-enantiomer (Fig. 2).

Kinetic analysis of MaTPS-SaH, MaTPS-Cin, and MaTPS-Tln revealed a three-fold difference in the calculated K_*m*_ values for GPP (11–31 μM). The sabinene hydrate synthase MaTPS-SaH showed the highest affinity for GPP, followed by terpinolene synthase MaTPS-Tln, and cineole synthase MaTPS-CinA has the lowest affinity for this substrate (Table 1). We also measured and compared the affinity of MaTPS-Tln for Mg^2+^ and Mn^2+^ ions as co-factors in the presence of GPP. MaTPS-Tln showed 90-fold greater affinity for manganese ions compared to magnesium ions (Table 1).

**Table 1:** Kinetics of three terpene synthase enzymes from *Melaleuca alternifolia* leaves

Enzyme	Substrate/cosubstrate	K_*m*_
MaTPS-SaH (sabinene hydrate synthase)	GPP	11 ± 2.0 μM
MaTPS-Cin (1,8-cineole synthase)	GPP	31 ± 6.26 μM
MaTPS-Tln (terpinolene synthase)	GPP	21 ± 3.18 μM
MaTPS-Tln (terpinolene synthase)	Mg^2+^	13 ± 0.14 mM
MaTPS-Tln (terpinolene synthase)	Mn^2+^	0.15 ± 0.01 mM

### The expression of the characterised genes in the six chemotypes in *M. alternifolia* (part 2)

The terpene profile of each sample was determined (data not shown) and three trees from each chemotype were selected for further study.

### Sequencing and mapping stats

We sequenced 424,141,194 reads at a read length of 150 bp (total 63.621 Gbp). After adaptor and low quality bases removal, the average read length was 139 bp. Individual samples varied from 11.8–36.8 m reads (average 23.6 m, median 21.7 m reads). The sum of the length of the 27 identified terpene synthase genes was 40,451 bp (average 1498 bp), indicating that some transcripts were not full length as the expected terpene synthase transcript is ca. 1800 bp long. On average, 81,897 reads mapped to the terpene synthase reference per individual (min 17,162; max 135,346, median 70,121) corresponding to 0.34% of the total reads. There was no effect of chemotype on the amount or proportion of reads that mapped to the terpene synthase transcripts. The largest difference was between chemotypes 2 and 3 which had 0.28 and 0.42% of their reads mapped against terpene synthase transcripts, respectively (student’s t-test *P* = 0.16). The expression level (FPKM) of each terpene synthase can be found in Additional file 2: Table S2.

**Fig. 3:**
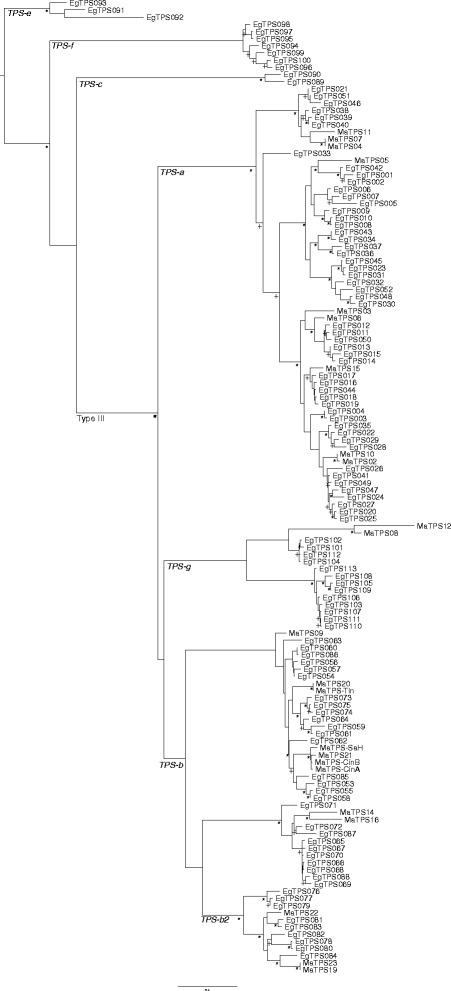
Maximum likelihood phylogeny of *M. alternifolia* terpene synthases including the four characterised genes and the 24 putative terpene synthase sequences, and the *E. grandis* terpene synthase gene family. The substitution model JTT + I + G + F was used, and 1000 bootstraps. + indicates bootstrap values >800 and * indicates bootstrap values >950. TPS subfamily labels are from [38]

We identified 27 putative terpene synthase sequences in the 18 foliar transcriptome libraries of six chemotypes of *M. alternifolia*. Each of the sequences has the conserved motifs common to all plant mono- and sesquiterpene synthases. Through sequence homology we found the characterised *MaTPS-Tln*, *MaTPS-CinA*, and *MaTPS-SaH*. Phylogenetic analysis with the terpene synthases of *E. grandis* [38] allowed us to group putative monoterpene synthases (TPS-b and TPS-g) separately from putative sesquiterpene synthases (TPS-a) (Fig. 3).

### Statistical analysis of relationships between terpene profiles and expression patterns (sPLS)

**Fig. 4:**
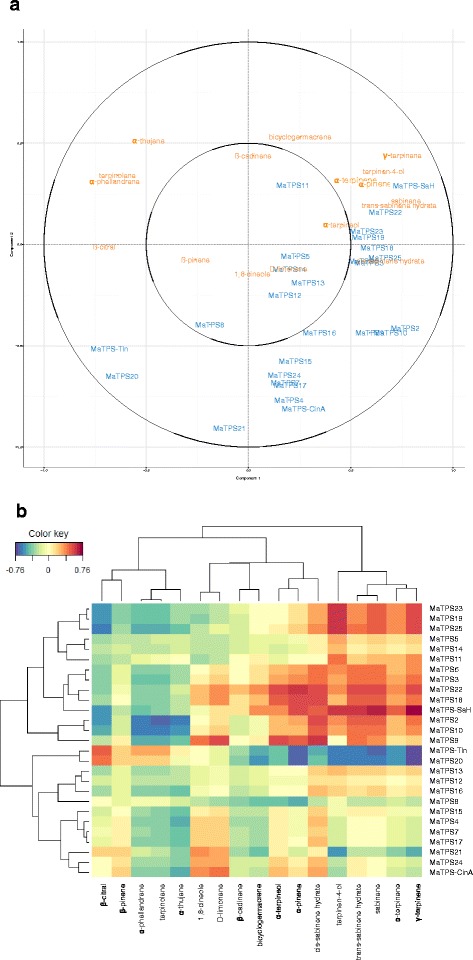
Results of the sPLS analysis between the concentration of the terpenes and the gene expression in *M. alternifolia* leaf samples. **a** Correlations between the first two principal components and each terpene proportion (orange text) or the gene expression (blue text) for variables selected with the sPLS analysis (see methods). Variables located in the same direction from the centre of the circle show a direct association. The further a variable is from the centre of the circle the stronger the correlations. **b** The correlation heatmap with a hierarchical cluster between the terpene matrix (x-axis) and the terpene synthase gene expression matrix (y-axis) using the first three principal components from the sPLS analysis. Blue cells indicate a negative correlation and red cells indicate a positive correlation with the intensity of the colour representing the strength of the correlation

In the circular correlation plot, variables that are most strongly associated are plotted in the same direction, and the greater the distance from the origin the stronger the correlation. In the plot generated using the full terpene matrix, the three terpenes that dominate the cardinal chemotypes were far apart in the circle (Fig. 4a). Terpinolene, α-phellandrene, β-citral, and α-thujene were grouped, as were terpinen-4-ol, sabinene, γ-terpinene, cis- and trans-sabinene hydrate, α-pinene, and α-terpinene; and 1,8-cineole and D-limonene. *MaTPS21* and *MaTPS-CinA* showed the highest correlation to 1,8-cineole (Fig. 4b). The genes most closely associated with the terpinolene group are *MaTPS-Tln* and *MaTPS20*. The genes most closely associated with the terpinen-4-ol group are *MaTPS-SaH*, *MaTPS25, MaTPS2, MaTPS10, MaTPS3, MaTPS6, MaTPS23*, and *MaTPS19.*

### The relationships between the terpene synthases of *M. alternifolia*

We identified 27 unique putative terpene synthase sequences in the RNA-Seq analysis. Three of these match the three of the characterised terpene synthases in this study. There is no sequence in the RNA-Seq that matches *MaTPS-CinB*. Of the remaining 24 putative terpene synthase sequences, 19 could be aligned to the sequences of the *Eucalyptus grandis* terpene synthase gene family [38] to determine which terpene synthase group they belong to (Fig. 3). The remaining five sequences had too many sequence ambiguities to align and so were excluded. The phylogeny generated here is very similar to the one reported by Külheim et al. [38]. We found representatives from each of the subfamilies of class III terpene synthases: TPS-a (angiosperm sesquiterpene synthases; *N* = 9), TPS-b (angiosperm monoterpene synthases; *N* = 11) and TPS-g (angiosperm acyclic monoterpene synthases; *N* = 2) expressed in the leaves of *M. alternifolia*. We did not find representatives of class I or II terpene synthases [38, 49, 50].

## Discussion

The overall aim of this study was to test the hypothesis that a sabinene hydrate synthase, a terpinolene synthase and a 1,8-cineole synthase are responsible for the production of six chemotypes in *Melaleuca alternifolia*, as proposed by Keszei et al. [13]. To do this, we first identified and characterised terpene synthases that each produce sabinene hydrate, terpinolene, and 1,8-cineole. Then we investigated the gene expression of each of these genes in leaves representing all chemotypes.

### Terpene synthase enzymes that produce terpinen-4-ol, terpinolene, and 1,8-cineole in the leaves of *M. alternifolia*

In this study, we isolated and characterised the enzymatic activity of four terpene synthase enzymes: MaTPS-SaH, MaTPS-Tln, MaTPS-CinA, and MaTPS-CinB, whose primary product is sabinene hydrate, terpinolene, 1,8-cineole, and 1,8-cineole, respectively (Fig. 1).

The two 1,8-cineole synthases have 96% amino acid identity to each other and most of the differences occur in the N-terminal domain, which does not form the catalytic pocket but it caps the active site when a substrate is bound [51] and contains the chloroplast targeting peptide of mono- and diterpene synthases. The tandem arginines, of the RRX_8_W motif, are thought to play a role in diphosphate migration during the formation of carbocation intermediates [52]. The dominant product of both enzymes is 1,8-cineole; however, they also produce smaller amounts of limonene, β-myrcene, sabinene, β-pinene, α-pinene, and α-terpineol (Fig. 1). This group of compounds, known as the ‘cineole cassette’ [53, 54], is produced by all characterised 1,8-cineole synthases reported from plants [19, 55–61].

There are four characterised terpinolene synthases from plants [62–65]. All four enzymes make terpinolene as their major product, with minor products including α-pinene, α-phellandrene, and limonene. The terpinolene synthase we have characterised from *M. alternifolia* also produces terpinolene and α-phellandrene, but not α-pinene and limonene (Fig. 1) and is therefore most like the terpinolene synthase from basil (*Ocimum bascilium*) [63].

We identified and characterised a sabinene hydrate synthase whose major product is (*1R,4R,5S*)-(*Z*)-sabinene hydrate. There are three characterised sabinene hydrate synthases from plants [66, 67] which each make a mixture of (*E*)- and (*Z*)-sabinene hydrate, along with very small amounts of other monoterpenes. Both (*E*)- and (*Z*)-sabinene hydrate have been described to convert non-enzymatically to terpinen-4-ol in planta [47, 48, 68].

We found that the sabinene hydrate synthase has the highest affinity for GPP (the precursor to monoterpenes) and the 1,8-cineole synthase (MaTPS-CinA) has the lowest affinity for GPP. The K_*m*_ values for all enzymes were in the range reported for other known terpene synthases [67, 69–77]. There are few studies that have compared the activity of terpene synthases with different co-factors, however it seems that magnesium and manganese are the most commonly used TPS co-factors in the plant kingdom [61, 69–77]. These studies suggest that monoterpene synthases have higher activity with manganese as a co-factor and sesqui- and diterpene synthases are more active with magnesium as a co-factor.

### Relationship between sabinene hydrate and 1,8-cineole synthases in *M. alternifolia*

Comparison of the three major monoterpene synthases suggested that terpinolene and sabinene hydrate synthases are more similar in their catalysis products (with eight products in common) than either is with 1,8-cineole synthase.

We suggest that sabinene hydrate synthases evolved from 1,8-cineole synthases in Myrtaceae. *MaTPS-CinA*, *MaTPS-CinB*, and *MaTPS-SaH* share 94–96% amino acid identity, yet the product profile of the MaTPS-SaH is very different to that of MaTPS-CinA and MaTPS-CinB. This suggests that the sequence similarity is due to shared ancestry rather than functional convergence. Additionally, we can amplify many different genes that encode 1,8-cineole synthases in Myrtaceae suggesting that there are multiple copies of 1,8-cineole synthases. In contrast, we have only ever amplified this single sabinene hydrate synthase despite examining multiple species of *Eucalyptus* and *Melaleuca* that have terpinen-4-ol as the dominant compound in the oil. If there are multiple sequences that share 94–96% amino acid identity and most of them produce 1,8-cineole, then we expect that the sabinene hydrate synthase arose by neofunctionalization of a 1,8-cineole synthase.

The products of the individual enzymes, each representing the most abundant monoterpene synthase transcript in the three cardinal chemotypes (Chemotypes 1, 2 and 5), match the biosynthetic groups proposed by Keszei et al. [13]. This lends support to our original hypothesis that these genes are sufficient to explain chemotypic variation in *M. alternifolia*.

### The three characterised genes are not sufficient to explain chemotypic variation in *M. alternifolia*

We used RNA-Seq to investigate the expression of terpene biosynthetic genes in the young leaves of six chemotypes of *M. alternifolia* from natural populations. We found that the most strongly associated terpenes fall within the biosynthetic groups proposed by Keszei et al. [13], which also matches the product profile of the characterised enzymes. Therefore, the chemical data suggests that main differences between the terpene profiles of different chemotypes could be explained by three terpene synthases. However, we found that all the characterised genes are expressed at similar levels in the leaves of each chemotype (average values from Additional file 2: Table S2 by chemotype).

Whilst the expression of the characterised terpene synthases is not sufficient to explain the maintenance of six chemotypes in *M. alternifolia*, the enzyme with the higher affinity for the shared substrate should produce more product. In other words, all else being equal, the terpene synthase with the lowest K_*m*_ value will produce the most terpene product if equal amounts of enzyme are present and all enzymes share the same substrate supply, since the reactions catalysed are irreversible [78, 79]. 1,8-Cineole is the dominant monoterpene found in chemotype 5 leaves, however the characterised 1,8-cineole synthase is not the most highly expressed terpene synthase in the transcriptomes of chemotype 5 individuals. Since the characterised monoterpene synthases are competing for the substrate FPP, we expect the MaTPS-CinA to have the lowest K_*m*_ and MaTPS-SaH to have the highest K_*m*_, to explain the difference between gene expression and phenotype. We found the opposite, so the substrate affinity of an enzyme doesn’t account for the disparity between gene expression and phenotype. Either other aspects of enzyme kinetics (k_cat_, V_max_) account for the patterns in terpene profile, or, more likely, other terpene synthase enzymes are involved.

### Other terpene synthases may play a role in the maintenance of chemotypic variation in natural populations of *M. alternifolia*

The putative terpene synthase sequences revealed in the RNA-Seq experiment share many similar features to other characterised terpene synthases [49, 50] and they align well with the *E. grandis* terpene synthase gene family [38], confirming their status as putative terpene synthase sequences (Fig. 3).

Since the three genes, *MaTPS-SaH*, *MaTPS-Tln*, and *MaTPS-Cin*, were not sufficient to explain the chemotypic variation, we expanded our search to other terpene synthases that could contribute to the foliar terpene profile. We used sparse partial least squares (sPLS) analysis to investigate the relationship between expression of the 27 terpene synthases and the foliar terpene profile (Fig. 4).

*MaTPS19* and *MaTPS23* are very similar to each other, and fall in the ocimene/isoprene group (TPS-b2, [38]). They are both synonymous with the characterised isoprene synthase from *M. alternifolia* [17], with all three sequences sharing >97% amino acid identity (data not shown). *MaTPS4* is likely to have a function similar to the two 1,8-cineole synthases (Fig. 3), which is supported by the sPLS analysis (Fig. 4), comparing the expression of each putative terpene synthase and the amount of each compound in the leaves.

The foliar concentration of the focus terpenes, terpinolene, 1,8-cineole, and terpinen-4-ol, correlates with the expression of *MaTPS-Tln*, *MaTPS-CinA,* and *MaTPS-SaH,* respectively, as well as with the additional terpene products of each characterised gene. However, there is also a strong correlation between the focal terpenes and the expression of uncharacterised terpene synthases (Fig. 4). These same enzymes also have strong correlations with other terpenes. The putative monoterpene synthase, *MaTPS20*, is predicted to encode another terpinolene synthase with very similar product profile to MaTPS-Tln. The putative monoterpene synthase *MaTPS25* is likely to encode an enzyme with a very similar product profile to that of MaTPS-SaH. The putative monoterpene synthase, *MaTPS21* is predicted to be a 1,8-cineole synthase with a similar product profile to MaTPS-CinA. Of particular note is *MaTPS9* a putative monoterpene synthase whose expression co-varies with 1,8-cineole, α-terpineol, (*E*)- and (*Z*)-sabinene hydrate but not with terpinen-4-ol in the foliar ethanol extracts. If these compounds dominate the product profile of *MaTPS9*, then this could be one of the first examples of an enzyme producing both 1,8-cineole and sabinene hydrate.

Other possible, but less likely explanations for the terpene profile not matching the expression of terpene synthases are: 1. post-transcriptional regulation, where the expression level of the gene doesn’t match the activity of the encoded protein, as shown in tissue cultures of Norway Spruce (*Picea abies*) [80]; 2. the compounds produced by some of these enzymes may not be stored in the leaf, but are released into the headspace of the plant, (e.g. Bustos-Segura et al. [11]); 3. the products from the expressed and characterised TPS enzymes could undergo further modifications, such oxidation by cytochrome P450 enzymes [81–85], methylation by *O*-methyl-transferases [86, 87] or conjugation to other metabolites to make new metabolites, as is the case for formylated phloroglucinol compounds found in *Eucalyptus* species [88, 89]. Each of these explanations require further investigation.

At first glance terpene chemotypes appear to offer relatively simple systems to investigate the molecular basis of ecologically important plant chemistry. However, the route to these chemical variations can be complex involving the expression of multiple genes within a framework of gene duplications and possible introgression from closely related species. Studies of chemotypic variation in non-model organisms, such as *Melaleuca alternifolia* and *Thymus vulgaris* offer a view of biodiversity that is easily missed and highlights the complexity of interactions in natural systems.

## Conclusions

We set out to test the hypothesis that three terpene synthases, a 1,8-cineole synthase, a terpinolene synthase and a sabinene hydrate synthase, are sufficient for the development and maintenance of six foliar terpene chemotypes in *Melaleuca alternifolia*. First, we discovered four novel genes in the leaves of *Melaleuca alternifolia*, that produce sabinene hydrate, 1,8-cineole and terpinolene. Then we used RNA-Seq to investigate the expression of these genes in the leaves of the six chemotypes. This analysis suggests that ‘chemotype’ is a more complex trait in *M. alternifolia* and the products of multiple terpene synthases, most of which remain uncharacterised, is the most likely explanation of the chemotypic patterns observed.

## Additional files

Additional file 1: Table S1. The amino acid similarity matrix (A), the amino acid identity matrix (B) and the cDNA sequence identity matrix (C) comparing the four full length sequences. (XLSX 8 kb)

Additional file 2: Table S2. The calculated FPKM values for the three characterised genes in foliar transcriptomes of *M. alternifolia* individuals representing the six chemotypes. Sample ID is an arbitrary sample number, Chemotype is defined by GC-MS analysis of foliar ethanol extracts of the leaves. (XLSX 16 kb)

## Abbreviations

FPKM: Fragments per kilobase of transcript per million mapped reads
FPP: Farnesyl pyrophosphate
GPP: Geranyl pyrophosphate
Km: Substrate concentration at which half of the maximum reaction velocity of an enzyme is achieved
RACE: Rapid amplification of complimentary ends
sPLS: Sparse partial least squares
SPME: Solid phase microextraction
TPS: Terpene synthase
